# Effect of music breathing, a program based on mindful breathing and music listening therapy for promoting sense of coherence in young people: study protocol for a randomized controlled trial

**DOI:** 10.1186/s13063-023-07645-x

**Published:** 2023-10-12

**Authors:** Winnie Lai-Sheung Cheng, Anson Chui-Yan Tang, Mark Cheuk-Man Tsang, Lokki Lok-Ki Wong, Dag Körlin

**Affiliations:** 1 School of Health Sciences, Caritas Institute of Higher Education, Hong Kong SAR, China; 2 School of Nursing, Tung Wah College, Hong Kong SAR, China; 3 School of Arts and Humanities, Tung Wah College, Hong Kong SAR, China; 4 School of Nursing and Health Studies, Hong Kong Metropolitan University, Hong Kong SAR, China; 5 IMAGEing: European GIM Trainings, Stockholm, Sweden

**Keywords:** Music therapy, Sense of coherence, Mental well-being, Stress, Mindfulness, Randomized controlled trial

## Abstract

**Background:**

The negative impacts of the COVID-19 pandemic on public health have affected people socially, psychologically, and physically. Young people particularly are having to adjust many aspects of their personal lives: including transitions to work, college, and independent living. Personal resources are important in mitigating stress and improve mental well-being during pandemic. Sense of coherence—an orientation to life—could be considered as a personal resource. Currently, a number of interventions have been developed to target the reduction of stress in young people. Little emphasis has been placed on developing sense of coherence to reduce stress and promote mental well-being among young people. Young people consider music as a preferred leisure activity and an important means of stress relief in their daily lives. However, little research concerning music therapy and sense of coherence exists.

**Methods:**

In the proposed randomized controlled trial, a sample of 290 young people (aged 18–30) will be recruited and allocated randomly into one of two groups: the experimental group and the control group. Participants in the experimental group will participate in a 6-week Music Breathing program that will include music listening and mindful breathing guided by a certified music therapist. Participants in the control group will receive a control condition for 6 weeks Mental Health Education Programme. The primary outcome of the study will be measured using Sense of Coherence Scale. The secondary outcomes will be measured using the Coping Self-Efficacy Scale, Difficulties in Emotion Regulation Scale, Mindful Attention Awareness Scale, Depression Anxiety Stress Scales, BBC Subjective Well-being scale, and salivary cortisol levels. Repeated measures analysis will be used to compare the outcomes between the two groups.

**Discussion:**

The results will inform practice in coping with stress through promoting sense of coherence. Individuals will benefit from the long-term effect of this intervention to enhance their sense of coherence to cope with stressful events and promote better mental well-being.

**Trial registration:**

ClinicalTrials.gov Identifier: NCT05655234. Registered on December 8, 2022.

## Administrative information

Note: the numbers in curly brackets in this protocol refer to SPIRIT checklist item numbers. The order of the items has been modified to group similar items (see http://www.equator-network.org/reporting-guidelines/spirit-2013-statement-defining-standard-protocol-items-for-clinical-trials/).

**Table:** 

Title {1}	Effects of Music Breathing, a program based on mindful breathing and music listening therapy for promoting sense of coherence in young people: study protocol for a randomized controlled trial
Trial registration {2a and 2b}.	ClinicalTrials.gov Identifier: NCT05655234. Registered on December 8, 2022.
Protocol version {3}	Ethical approval of this project was granted by the Research Ethics Committee, Tung Wah College [Ethics application number: REC2022122] and subsequently by the Research and Ethics Committee of Caritas Institute of Higher Education [HRE230202] on 26/6/2023 after the transfer of the project from Tung Wah College to Caritas Institute of Higher Education
Funding {4}	This project is funded by the Research Grants Council for the Competitive Research Funding Schemes for the Local Self-financing Degree sector under the Faculty Development Scheme [Reference number: UGC/FDS17/H03/22 (reassigned number after the project transfer: UGC/FDS17(11)/H03/22]. The funder provided financial support for the publication fee, dissemination of research findings, purchase of study materials and supporting the salary of the research staff. The funder involves only monitoring adherence to the study protocol.
Author details {5a}	^1^School of Health Sciences, Caritas Institute of Higher Education, Hong Kong SAR, China. ^2^School of Nursing, Tung Wah College, Hong Kong SAR, China. ^3^School of Arts and Humanities, Tung Wah College, Hong Kong SAR, ^4^School of Nursing, Hong Kong Metropolitan University, Hong Kong SAR, China, ^5^IMAGEing: European GIM Trainings, Sweden
Name and contact information for the trial sponsor {5b}	School of Health Sciences, Caritas Institute of Higher Education, Hong Kong SAR, China. 2 Chui Ling Lane, Tseung Kwan O, New Territories, Hong Kong [The project was transferred from Tung Wah College to Caritas Institute of Higher Education on 24/10/2022]
Role of sponsor {5c}	The sponsor does not have a role in the design of the study, data collection, analysis, and interpretation, and in writing the manuscript.

## Introduction

### Background and rationale {6a}

Stress arises when individuals perceive that they are powerless to cope with the demands placed on them [1]. Chronic stress may lead to physiological [2] and psychological illnesses [3] and impact adversely on the quality of life [4]. Young people experience many challenges and stressors during the transition from adolescence to adulthood. Currently, these challenges have been heightened by the global effects of the COVID-19 pandemic, which increases concerns associated with academic challenges, financial burdens, relationships, and uncertainty about the future [5].

Coping with these stressful events has never been more imperative in order to maintain a healthy mental state in young people. Coping is a constantly evolving cognitive and behavioral effort to manage situations appraised as stressful [6]. It involves both emotion-focused coping (regulating the emotional responses to stressful events) and problem-focused coping strategies (managing the problems causing stress). The type of coping strategy is influenced by the evaluation of options for coping, referred to as “secondary appraisal” [6]. Secondary appraisal involves the evaluation of personal resources that can be used to manage situations. The cognitive appraisal of one’s resources including both internal and external environmental resources is important for that individual to determine coping strategies in the form of behavioral actions. Individuals with personal resources that can facilitate positive coping strategies and behaviors are more adaptive to stressful situations.

Sense of coherence (SOC) can be considered a personal resource that is important to resolve stress and tension [7]. SOC is defined as an enduring tendency of individuals to view life as comprehensible, manageable, and meaningful [8]. It is a central component of the Salutogenic Model which places a great emphasis on the role of personal resources in promoting well-being and focuses on positive qualities [8]. SOC could be a component in the process of secondary appraisal that influences the coping options.

Previous studies showed that SOC is associated with quality of life, health behavior, and mental health in young people [9]. Furthermore, SOC has a mediating effect between perceived stress and health status in young people [10], and a better SOC is associated with positive coping styles in university students [11]. Therefore, young people with a strong SOC are more likely to perceive stressful situations as less threatening and can facilitate the use of coping strategies to manage stressors and ultimately lead to better mental well-being. Though SOC is considered as a stable attribute, studies have shown that it can be strengthened in adulthood [12]. Hence, interventions that enhance SOC as a personal resource in the cognitive process of stress appraisal and coping could be especially helpful for young individuals.

Researchers have studied the effects of various interventions to increase SOC, but the findings have been mixed. For instance, a recovery program in ICU patients did not result in significant differences in SOC and quality of life [13], whereas a resistance training program for older adults [14] and a self-management program for retired individuals with chronic diseases showed significant improvement in SOC [15]. Additionally, a scoping review by Suárez Álvarez et al. [16] reported methodological limitations in most of the SOC-related interventional studies.

While a number of interventions have been developed to reduce stress in young people, there has been little emphasis placed on promoting SOC to alleviate stress and improve mental well-being. Music therapy, among other health promotion strategies, has been widely used as a coping mechanism to reduce stress [17]. The ability of music therapy to regulate emotion has been well-known [18]. Through emotion regulation, individuals are able to maintain a comfortable state of arousal which allows them to respond and react to stressful situations flexibly [18]. Music therapy can regulate emotion leading to better psychological well-being [19], improve psychological stress-related symptoms (e.g., anxiety, nervousness, restlessness), promote personal growth leading to psychological and physiological health [20, 21], and enhance subjective happiness [22]. However, little research has so far been conducted to evaluate the effects of music therapy on SOC.

Alternatively, mindfulness-based interventions have been widely used to reduce psychological stress [23, 24]. Breathing is often used as a focus of concentration during the delivery of mindfulness-based interventions [24]. Studies have shown that combining mindful breathing techniques with music therapy can help individuals develop awareness of their experiences, regulate their emotions, and cope with stress in a more adaptive manner [25]. It has also been found to reduce pain and anxiety and improve sleep quality in patients with osteosarcoma [26] and alleviate anxiety and stress among healthy young adults [27]. These studies have primarily focused on the effects of music therapy and mindful breathing in reducing stress and anxiety and improving emotional regulation, but the mechanism of stress and coping through the development of personal resource—SOC—have not yet been explored. As positive coping skills are crucial for proactively managing stressful situations, further research is needed on the role of personal resources in coping with stress using music listening therapy and mindful breathing.

Young people often consider music as a preferred leisure activity and an important means of stress relief in their daily lives [28]. Therefore, a well-designed therapeutic program that uses music as a medium may be more appealing to young individuals and encourage their participation. The proposed study aims to integrate the practice of mindful breathing and music listening through a Music Breathing (MB) program, with the goal of promoting personal resources, reducing stress, and improving mental well-being in young people.

## Objectives {7}

The purpose of this project is to evaluate the effectiveness of the MB program in promoting SOC in young people, using a randomized controlled trial design. The proposed objectives are as follows: (i) to examine the effect of the MB program on promoting personal resources in young people, (ii) to examine the effect of the MB program on reducing stress-related symptoms in young people, and (iii) to examine the effect of the MB program on promoting mental well-being in young people. The hypotheses for the proposed study are as follows:

- H1: Participants who receive the MB program will demonstrate better SOC, emotion regulation, coping self-efficacy, and mindfulness than those in the control condition.
- H2: Participants who receive the MB program will exhibit reduced stress-related symptoms and a better physiological outcome, i.e., salivary cortisol levels compared to those in the control condition.
- H3: Participants who receive the MB program will have better mental well-being than those in the control condition.
- H4: There is a positive association between SOC, coping self-efficacy, emotion regulation, and mindfulness.

## Trial design {8}

**Fig. 1:**
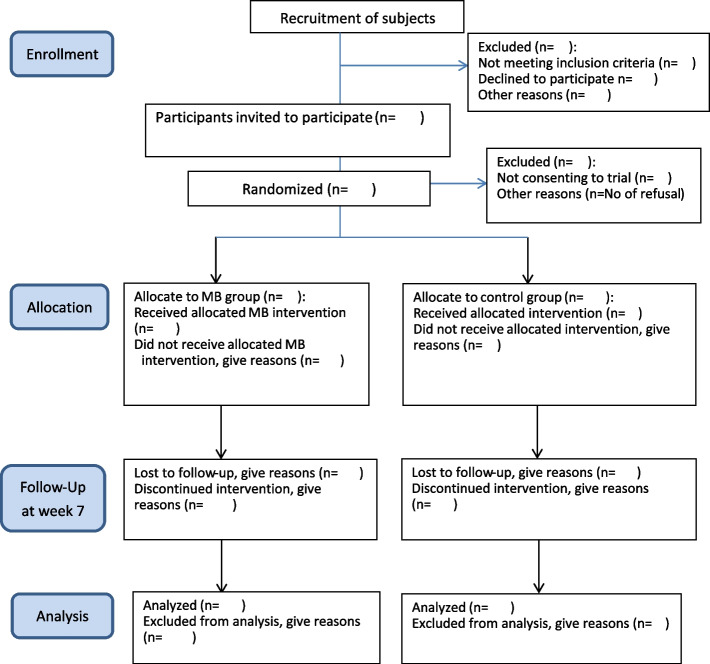
Study flow diagram

We will conduct a two-parallel-group, superiority, randomized controlled trial (RCT) to evaluate the effects of the MB program. Participants will be allocated to either the intervention or control group in a 1:1 ratio using computer-generated random numbers (Excel 2007, Microsoft, Redmond, WA, USA) after providing their consent to participate. Participants in the intervention group will receive a 6-week MB program, whereas those in the control group will receive a 6-week placebo control program. Data will be collected at three time points, i.e., before the intervention (T0), after the intervention (week 6; T1), and 1-month follow-up (week 10; T2), except for salivary cortisol levels, which will only be assessed at T1. Figure 1 illustrates the study flow.

## Methods: participants, interventions and outcomes

### Study setting {9}

The study will be conducted on the campus of the principal investigator (WC). Participants will be recruited from universities, higher education institutes, and district centers situated in various districts of Hong Kong such as Tsang Kwan O, Yau Tsim Mong. Invitations will be extended through various channels, including emails, social media, leaflets, and posters.

### Eligibility criteria {10}

Participants will be eligible for recruitment if they meet the following inclusion criteria: young people aged 18 to 30, a moderate level of stress (Perceived Stress Scale (PSS-10) score ≥ 14–26/40), and a liking for music. Participants diagnosed with an acute mental disorder, under the influence of drugs that affect the nervous system, or with previous experience with music-evoked imagery and meditations where music is played will be excluded.

### Who will take informed consent? {26a}

All participants will be required to provide written informed consent, which will be obtained by a research assistant trained by the research team.

### Additional consent provisions for collection and use of participant data and biological specimens {26b}

The consent form has included a statement of agreement for the collection of salivary samples.

## Interventions

### Explanation for the choice of comparators {6b}

The two comparators are Music Breathing (MB, experimental group) and Mental Health Education (MHE, control group). The comparator aligns with the aim of the study, which is to determine the effectiveness of the effect of the 6-week MB program on the SOC among young adults.

### Intervention description {11a}

The MB program is the integration of practicing mindful breathing and music listening. Instrumental classical music that is highly repetitive, in terms of melody, harmony, and rhythm will be used.

The MB program consists of four stages: stage 1—silent breathing for grounding (SB), stage 2—music breathing for grounding (MBG), stage 3—music breathing for modulation (MM), and stage 4—music breathing for working (MW).

- In SB, grounded breathing is practiced without music; it comprises discovery breathing and silent breathing as grounding activities. Discovery breathing involves breathing as spontaneously as possible, without direction. Silent breathing is practiced in two forms: triangular breathing and biphasic breathing. It involves exhaling for 4 counts, with the intention of staying in the breathing center as the holding position for another 4 counts, then inhaling for another 4 counts. This pattern is repeated for approximately 3 min. Biphasic breathing is regarded as normal breathing with small breathing volume like a sphere approximately the size of an orange around the breathing center. The SB aims to establish an image of a secure small breathing center which aims to facilitate the parasympathetic system where alarm responses are counteracted.
- In MBG, a grounding stage with music, a suitable musical piece with a predictable beat is introduced to support the rhythm and pace of meditative breathing.
- In MM, a modulation stage, music pieces with greater dynamics, and variations are used, and the breathing space is continuously modulated to follow and contain the music.
- In MW, a working stage, more forceful music is used, which requires the individuals to vary their breath volume with faster breathing rhythm to adjust to the higher levels of arousal and modulation within their Window of Tolerance.
- After completing the silent breathing and music breathing practices, the participants will draw pictures of their body images of breathing space and center. The form of the imagined breathing space expressed in the drawing and the meaning and content of the experience will be explored through a process facilitated by the therapist. The therapist’s understanding of the participants’ difficulties and progress will guide the next step, particularly the selection of music for the next sessions.

The experimental group will receive the MB program in 6 weekly sessions (each session lasts 2 h in a group of 6–8 participants) delivered in a confined and quiet room on the campus, plus a session of home practice on a weekly basis. The study intervention will be delivered by a certified music therapist with at least 25 h of MB program training by the trainer (i.e., the author, DK). The detail of the study intervention is presented in Table 1.

**Table 1:** Study intervention

Session	Activities (2 h)	Weekly home exercise
1	a) Concept of mindfulness b) Discovery breathing c) Draw the mental image of the practice of discovery breathing d) Triangular breathing e) Silent Breathing (SB) f) Draw the mental image of the practice of SB g) Debriefing - Share and process the experience	- 5-min practice - Practice triangular breathing and SB and draw the mental image of the practice of SB
2	a) Sharing and debriefing of the mental image drawn at home b) SB practice and drawing to the mental image of SB practice and debriefing c) Music Breathing (MB) d) Draw the mental image of MB with debriefing e) Debriefing - Share and process the experience	- 8-min practice - Practice SB, MB (use music in the 2nd session) and the mental image of the practice of MB
3	a) Sharing and debriefing of the mental image drawn at home b) SB practice and drawing to the mental image of SB practice and debriefing c) Music Breathing (MB) d) Draw the mental image of MB with debriefing e) Debriefing - Share and process the experience	- 11-min practice - Practice SB, MB (use music in present and previous sessions), and draw the mental image of the practice of MB
4	a) Sharing and debriefing of the mental image drawn at home b) Music Breathing (MB) c) Draw the mental image of MB with debriefing d) 30-s Music listening with breathing e) Discourse on the effects of each piece of music on self f) Debriefing - Share and process the experience	- 14-min practice - Practice SB, MB (use music in present and previous sessions), and draw the mental image of the practice of MB
5	a) Sharing and debriefing of the mental image drawn at home b) Music Breathing (MB) - Perform breathing with listening to music pieces selected by therapist from the pre-arranged list c) Draw the mental image of MB with debriefing d) 30-s Music listening e) Debriefing - Share and process the experience	- 17-min practice - Practice SB, MB (use music in present and previous sessions), and draw the mental image of the practice of MB
6	a) Sharing and debriefing of the mental image drawn at home b) Music Breathing (MB) c) Draw the mental image of MB with debriefing d) Movement to the music e) Debriefing - Share and process the experience f) Closing activities/ritual	- 20-min practice - Practice SB, MB (use music in present and previous sessions), and draw the mental image of the practice of MB

#### Home practice

Participants will be asked to practice mindful breathing, music listening, and drawing images according to a weekly schedule during the 6-week intervention. They will be required to record the frequency and duration of their home practice. During the orientation in the first week, participants will be asked to work out a plan for implementing home practice and complete an online schedule for the subsequent weeks. The research team will monitor the participants’ progress and provide motivation and encouragement to ensure continued home practice.

Participants in the control group (6–8 participants per group) will receive a 30-min Mental Health Education Programme (MHE) in the first meeting (week 1) and second meeting (week 6). The content of the MHE will be adopted from the Student Health Service, Department of Health, Hong Kong SAR. The MHE is comprised of the following topics:

What is Stress? https://www.studenthealth.gov.hk/english/health/health_ph/health_ph_stress.html);

Tips on Being Joyful** (**https://www.chp.gov.hk/archive/joyful/en/contentsab5e.html?id=40);

Breathing Exercise practice (https://www.studenthealth.gov.hk/english/emotional_health_tips/eht_re/eht_re.html); and Music listening.

After the first meeting, participants in the control group will be provided with a take-home kit containing a fact sheet with information from the health talk and a video link to the breathing exercises and musical pieces. They will be instructed to practice breathing exercises and listen to the musical pieces at home. Weekly reminders via WhatsApp message and/or email will be sent to enhance compliance. During the second meeting, participants will receive a revision of the MHE and have the opportunity to share their stress management experience with the other group members.

### Criteria for discontinuing or modifying allocated interventions {11b}

This is a low-risk study, and there are no specific criteria for discontinuing or modifying allocated interventions for participants. Participants will be informed that they have the right to stop participating in the study for any reason at any time without consequence and without having to give further explanation. Any discontinuation will be documented for the individual participant.

### Strategies to improve adherence to interventions {11c}

To increase compliance, participants will receive weekly reminders via WhatsApp messages or email to ensure practice at home. Reminders via WhatsApp and email will be sent to the participants before each session to enhance their adherence to the interventions; participants will also receive email and WhatsApp reminders if they have not completed the questionnaires or returned the saliva sample.

### Relevant concomitant care permitted or prohibited during the trial {11d}

Participants will be allowed to continue their usual practices throughout the study, and no concomitant care will be either permitted or prohibited during the trial.

### Provisions for post-trial care {30}

Not applicable. This is a low-risk trial and there will be no adverse effects.

### Outcomes {12}

Primary and secondary measures will be collected before-intervention (T0), after-intervention (week 6; T1), and 1-month follow-up (week 10, T2) via a self-administered questionnaire. The salivary cortisol levels will be collected at T1.

### Primary outcome

#### Sense of coherence

The 13-item Chinese version of the Sense of Coherence Scale (C-SOC-13) [29] will be used to measure the comprehensibility, manageability, and meaningfulness of the participants’ lives [8]. This scale is scored on a 7-point semantic scale with two anchoring phrases, ranging from 1 (very poor) to 7 (very strong). The higher mean score represents a greater sense of coherence. The original SOC-13 was shown to have high internal consistency (Cronbach’s α = 0.74–0.91) [8].

### Secondary outcomes

#### Coping self-efficacy

The Coping Self-Efficacy Scale (CSES) is used to measure a person’s confidence in their ability to cope effectively with stress [30]. It is a 26-item scale with three subscales: (1) use problem-focused coping, (2) stop unpleasant emotions, and (3) seek support from friends and family. The CSES has strong reliability (α = 0.79–0.92) and concurrent validity. It is rated on an 11-point scale on which the participant believes they could perform the behavior. The anchor points range from 0 (cannot do at all) to 10 (certain can do). Higher scores indicate higher levels of coping self-efficacy.

#### Emotion regulation

The Difficulties in Emotion Regulation Scale (DERS) is used to assess the ability to regulate emotions [31]. It contains a total scale score (36 items) and six factors including nonacceptance of emotional response, difficulties in engaging in goal-directed activity, impulse control difficulties, lack of emotional awareness, limited access to emotion regulation strategies, and lack of emotional clarity. Items are measured on a 5-point Likert-type scale from 1 (almost never) to 5 (almost always), with higher values indicating difficulty in emotion regulation.

#### Mindfulness

The 15-item Mindful Attention Awareness Scale (MAAS) (Chinese version) will be used to assess attentiveness to and awareness of experiences in the present moment [32]. This scale is scored from 0 (almost always) to 6 (almost never); a higher mean score reflects a higher level of mindfulness. The internal consistency of the scale is good (coefficient of reliability > .82).

#### Depression, anxiety and stress

The 21-item Depression Anxiety Stress Scales (DASS-21) (Chinese version) comprise 21 statements on three components: (a) depression, (b) anxiety, and (c) stress [33]. The scales are scored using a 4-point Likert scale with response options from 0 (does not apply to me at all) to 3 (applies to me very much, or most of the time). The internal consistency has been demonstrated to be good (Cronbach’s α values: depression = .88, anxiety = .75, and stress = .81).

#### Subjective general well-being

The 24-item BBC Subjective Well-being Scale (BBC-SWB) will be used to measure the participants’ subjective experiences of general well-being [34]. It measures three components: psychological well-being, physical health and well-being and relationships with good internal consistency (Cronbach’s α = .94) and concurrent validity (r = .640– .80) [34]. The scale is scored using a 5-point Likert-scale.

#### Salivary cortisol

Salivary cortisol levels (sCort) will be used as a biomarker of psychological stress. sCort is considered a reliable measure of hypothalamus pituitary adrenal axis (HPAA) adaptation to stress and correlates well with plasma cortisol [35]. Average sCort levels in healthy subjects are 5.52–28.92 nM/l in the morning and 1.10–11.32 nM/l in the afternoon [36]. sCort is stable for several days, non-invasive, and easily obtained without adding stress to subjects [36], so is suitable for this study in which the participants are required to provide multiple samples at home.

#### Demographic information

A self-developed questionnaire will be used to collect demographic information about the participants, including age, gender, educational level, religious beliefs and practices, socioeconomic status, and coping behavior. The latter will be assessed using the Brief Cope Inventory (COPE) [37].

### Participant timeline {13}

The participant timeline is presented in Table 2.

**Table 2:**
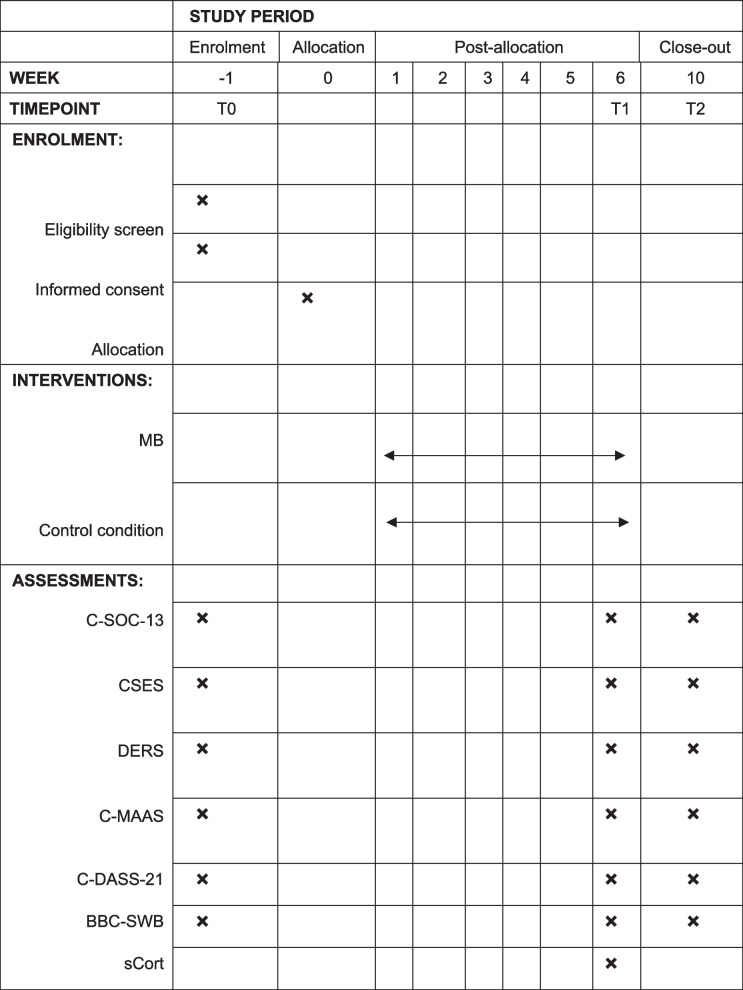
Participant timeline

*MB* Music Breathing Programme,* C-SOC-13* The 13-item Chinese version of the Sense of Coherence Scale, *CSES* Coping Self-Efficacy Scale, *DERS* The Difficulties in Emotion Regulation Scale, *C-MAAS* The Chinese version of the 15-item Mindful Attention Awareness Scale, *C-DASS-21* the Chinese version of the 21-item Depression Anxiety Stress Scales, *BBC-SWB* The 24-item BBC Subjective Well-being Scale,* sCort*, Salivary cortisol level

### Sample size {14}

The estimated sample size has been calculated using the statistical package G*Power 3.1.9.4 (version 2019) for a multivariate analysis of variance (MANOVA) according to the effect size (Cohen’s *d*) (between 0.43 and 0.59) of anxiety in a previous study on group music-guided imagery [38]. When converting to a repeated measure (Cohen’s *f*), the effect size is considered moderate (*f* = 0.25), with a type I error rate of 5% (2-sided) and assuming a correlation of 0.5 between repeated measures. To achieve 90% power to measure outcome three times in two groups, 232 subjects (*n* = 116 per group) will be required [39]. Assuming a 20% attrition rate [40], the trial will require at least 145 participants starting in each arm. We target to complete the recruitment process within 20 weeks; therefore, an average rate of recruitment of about 14 subjects per week is anticipated. It might be possible to shorten the recruitment period if the threat posed by the pandemic weakens.

### Recruitment {15}

The study aims to recruit participants from a diverse range of locations, including universities, higher education institutes, and district centers situated in in various districts of Hong Kong such as Tsang Kwan O, Yau Tsim Mong, using various methods such as seminars, social media, leaflets, and posters. To ensure efficient recruitment, the study team members will assist in reaching out to potential participants. Additionally, the coordinator of local community centers for young people will be contacted to aid in recruitment efforts.

To reach a wider audience, promotional posters, leaflets, and a website containing information about the study and contact details of the study team will be created. The website page will be promoted on social media platforms such as Meta, and the website link, along with electronic and printed posters, will be distributed to community centers and tertiary institutions to reach potential participants.

Promotional seminars will also be conducted to attract interested participants and provide them with more information about the study. Potential participants who are interested in participating can contact the study team for further information and undergo a screening process to determine their eligibility. Eligible participants will be notified of the study's procedures and any further arrangements that need to be made.

## Assignment of interventions: allocation

### Sequence generation {16a}

The participants will be randomly allocated to the intervention or control group in a 1:1 ratio after obtaining informed consent. The randomization process will involve computer-generated random numbers using Microsoft Excel 2007 (Microsoft, Redmond, WA, USA).

### Concealment mechanism {16b}

The random allocation will be conducted by a research assistant who will not be involved in the intervention and data analysis. The allocation sequence will be stored in a secure folder on a secure server until interventions are assigned and will only be accessible to the research assistant who created the sequence.

### Implementation {16c}

The research assistant who generated the allocation sequence will assign a group label to each participant based on the generated sequence. The study team member will only have access to the assignments for participants who are ready to enroll and will not be able to view the future allocation sequence.

## Assignment of interventions: blinding

### Who will be blinded {17a}

In this study, the participants will be blinded to their group assignment. The interventionists and research team members will have knowledge of the group assignment after enrolment. A research team member who is blinded to the treatment assignments will perform the data analysis. Participants in both the experimental and control groups will be provided with information regarding the study procedures of MB and MHE program, respectively, after completing the baseline questionnaire.

### Procedure for unblinding if needed {17b}

There will be no unblinding procedure in this study.

## Data collection and management

### Plans for assessment and collection of outcomes {18a}

Data will be collected from both groups at three time points: before-intervention (T0), after-intervention (week 6; T1) and 1-month follow-up (week 10; T2), except the salivary cortisol levels which will be assessed at T1. The questionnaires will be completed by participants using an online data collection system which will be constructed to force answering to prevent data missing. Saliva samples will be collected using Cortisol Salivary Test kits and analyzed using a commercially available enzyme-linked immunoassay (eNano Health). Participants will be asked to provide 2 samples: 0700–0800 h and 1600–1700 h to avoid meals within 60 min of providing each sample and to avoid caffeine-containing food and drink during the 2 days of sampling. Participants will be instructed to keep the samples in an envelope and send them to the laboratory for analysis. The results of the samples will be reported from the laboratory and the data will be entered into the database by a research assistant who will not be involved in the intervention and data analysis.

### Plans to promote participant retention and complete follow-up {18b}

To enhance participant adherence, a research assistant will send regular reminders to complete the questionnaires and encourage adherence to the study protocol for home practice. Moreover, to increase motivation to remain in the study, participants will receive a beverage coupon valued at HK$50 (approximately US$6) upon completion of the post-intervention questionnaire.

### Data management {19}

The research team will use a web-based data collection system to collect data. Participants will not input their names or any identifying information when accessing the questionnaires but will be assigned a unique identifier upon completion. After each round of data collection, the data will be downloaded and saved on the secure network with password protection, ensuring that only authorized users (e.g., the research team members) will have access to the dataset.

### Confidentiality {27}

All data provided will be kept confidential and only be used in our research. All information collected will be input into the computer and encrypted. Copies of the questionnaire after data entry will be stored in a locked cabinet that can only be accessed by authorized users. All data and information will be used for research purpose only. Information provided will be identifiable by codes known only to the research team The procedure will be audio-recorded and stored electronically and held under password protection and will be destroyed after the study. Only the research team members who are responsible for monitoring and/or audit of the study to ensure that the research is complying with applicable regulations will be given access to data.

### Plans for collection, laboratory evaluation and storage of biological specimens for genetic or molecular analysis in this trial/future use {33}

Biological specimens will be collected from saliva using a Cortisol Salivary Test kit and analyzed using a commercially available enzyme-linked immunoassay (eNano Health). To ensure proper sample collection, participants will receive user video instructions that provide guidance on the collection process. This self-collection method can be safely and easily performed by participants in the comfort of their own homes. Salivary cortisol is stable at room temperature for several days. Participants will be asked to provide 2 samples: 0700–0800 h and 1600–1700 h, to avoid meals within 60 min of providing each sample and to avoid caffeine-containing food and drink during the day of sampling. After collection, participants will be instructed to place the samples into the envelope, seal the envelope, and send it to the laboratory for analysis on the day of collection. There will be no storage for ancillary studies.

## Statistical methods

### Statistical methods for primary and secondary outcomes {20a}

The statistical analysis will be performed in accordance with the study design, RCT with an intention-to-treat analysis using SPSS, Version 26. The baseline characteristics of the participants in the two arms will be compared to identify any clinically meaningful differences at baseline and at each time point using chi-square test (for nominal data), Mann–Whitney *U* test (for ordinal or non-normality data), or *t*-test (for normality data). Any variables considered to be significantly different will be further evaluated (e.g., included as covariates in the statistical analyses) and adjusted to account for their potential confounding effects on the outcomes. To measure both the primary and secondary outcomes, we will obtain repeated measures of multiple outcomes in the two groups, and the data will be analyzed using generalized estimating equation (GEE), with adjustment of the baseline outcome variables to test for between-group differences. Correlation analyses will be performed to determine the relationships among the dependent variables. Regression analyses and ANOVA will be conducted to assess significant differences in the effects of the demographic variables on each outcome variable. Repeated measures of ANOVA will be performed to explore the levels of sCort differing within subjects and between groups; between the two sampling days (day) and across the five sampling times (time). Path analyses will be conducted to test the mediating effects between stress-related symptoms and personal strength and emotional regulation.

### Interim analyses {21b}

There will be no planned interim analyses for this study.

### Methods for additional analyses (e.g., subgroup analyses) {20b}

Sub-group analyses will be conducted according to categorizations such as gender, coping style, and religion.

### Methods in analysis to handle protocol non-adherence and any statistical methods to handle missing data {20c}

We will interpret the data using intention-to-treat. Participants who do not adhere to the protocol are still included in the analysis. Missing values will be handled using an appropriate imputation method.

### Plans to give access to the full protocol, participant-level data and statistical code {31c}

The datasets analyzed during the current study and statistical code are available from the corresponding author on reasonable request, as is the full protocol.

## Oversight and monitoring

### Composition of the coordinating center and trial steering committee {5d}

There is no coordinating center for this trial. The trial will be led by the principal investigator (i.e., the author, WC), who will oversee all research activities and reporting requirements, supported by research team members (i.e., AT, MT, LW, DK) who form a steering committee independent of the sponsor and competing interests. The steering committee will be responsible for executing the study protocol, methodology, data monitoring, and statistical analyses. The research team comprises a certified psychotherapist and certified psychiatrist (DK), who will provide training to the MB interventionists and at least three supervision sessions to ensure the treatment fidelity; three academics in the nursing discipline (WC, AT, LW), who will contribute to recruitment efforts, control condition implementation, and coordinate data collection; and a statistician (MT), who will contribute to statistical analyses of this randomized controlled trial to evaluate the effectiveness of MB.

In addition to the steering committee, there is a project implementation team which includes two research assistants to support the day-to-day operation of the trial. The principal investigator will have weekly meetings with them to discuss the status of the project, separate from the steering committee meetings.

### Composition of the data monitoring committee, its role and reporting structure {21a}

The steering committee will also have the responsibility of monitoring the data in addition to supervising the trial and ensuring adherence to the study protocol.

### Adverse event reporting and harms {22}

Due to the low-risk nature of the trial, no adverse events are anticipated. In case of any adverse events are identified, the principal investigator will evaluate the situation and report to the research and ethics committee promptly.

### Frequency and plans for auditing trial conduct {23}

The principal investigator will be responsible for conducting and monitoring the trial. The steering committee will meet monthly to receive updates on the project.

### Plans for communicating important protocol amendments to relevant parties (e.g., trial participants, ethical committees) {25}

All minor modifications of the protocol will be approved by the research and ethics committee. Major amendments will be approved by the funding body. Changes will be updated on the trial registry (ClinicalTrials.gov).

### Dissemination plans {31a}

The results of this trial will be presented at local and/or international conferences and published in peer-reviewed journals. Additionally, the trial results will be reported to ClinicalTrials.gov.

## Discussion

The call to investigate effective strategies for stress management and improved mental well-being among young people has been prominent in this decade, particularly during the global pandemic period. The findings from the proposed study are expected to provide support for the potential benefits of a therapy option that integrates mindful breathing and music therapy with the intent to engender SOC in coping with stress in young people. The results will add knowledge of SOC to the stress and coping process and support the advancement of stress reduction-related knowledge. The findings will advance the level of professional knowledge, thus supporting researchers, healthcare professionals, and policymakers who plan, implement, and evaluate public health services and improve teaching and learning. This therapy option provides an opportunity for young people to learn the techniques and benefit from the long-term effect of this intervention. In the long run, young people will have an improved ability to cope with stressors in their everyday lives and will experience fewer psychological burdens and better health. For young people, improved mental well-being can facilitate a healthy transition into adulthood and should yield promising results throughout their lives.

The recruitment of interventionists with expertise in MB is challenging due to the limited number of music therapists trained in this area. The resulting scarcity of available interventionists may constrain the delivery of intervention sessions. Additionally, the recruitment process may be prolonged due to the socially active nature of young people and their engagement in learning and work, despite the research team's promotional efforts. To ensure the timely completion of the project, interventionists will be asked to offer timeslots in the evenings and on weekends to accommodate participants’ schedules. While the questionnaires will be accessible online, there is a possibility that some participants may not complete the questionnaires within the allocated timeframe. To mitigate this potential challenge, reminders will be sent via email and WhatsApp to encourage participants to complete the questionnaires promptly.

## Trial status

Recruitment of participants will begin in August 2023 and is expected to be completed by May 2024.

## Data Availability

The data will be available upon reasonable request.

## Abbreviations

BBC-SWB: BBC subjective well-being scale
COPE: Brief Cope Inventory
CSES: The Coping Self-Efficacy Scale
DASS-21: The 21-item Depression Anxiety Stress Scales
DERS: Difficulties in Emotion Regulation Scale
MAAS: The 15-item Mindful Attention Awareness Scale
MB: Music Breathing
MM: Music breathing for modulation
MBG: Music breathing for grounding
MHE: Mental Health Education
MW: Music breathing for working
PSS: Perceived Stress Scale
RCT: Randomized controlled trial
SB: Silent breathing for grounding
sCort: Salivary cortisol levels
SOC: Sense of coherence
